# Diagnostic Accuracy of a Prototype Point-of-Care Test for Ocular *Chlamydia trachomatis* under Field Conditions in The Gambia and Senegal

**DOI:** 10.1371/journal.pntd.0001234

**Published:** 2011-08-02

**Authors:** Emma M. Harding-Esch, Martin J. Holland, Jean-François Schémann, Sandra Molina, Isatou Sarr, Aura A. Andreasen, Chrissy h. Roberts, Ansumana Sillah, Boubacar Sarr, Edward F. Harding, Tansy Edwards, Robin L. Bailey, David C. W. Mabey

**Affiliations:** 1 London School of Hygiene and Tropical Medicine, London, United Kingdom; 2 Medical Research Council Laboratories, Fajara, Banjul, The Gambia; 3 Institut de Recherche pour le Développement (IRD), Dakar, Sénégal; 4 National Eye Care Programme, Ministry of Health, Banjul, The Gambia; 5 Programme National de Lutte contre la Cécité, Ministère de la Santé, Dakar, Sénégal; 6 Ely, United Kingdom; University of California San Francisco, United States of America

## Abstract

**Background:**

The clinical signs of active trachoma are often present in the absence of ocular *Chlamydia trachomatis* infection in low prevalence and mass treated settings. Treatment decisions are currently based on the prevalence of clinical signs, and this may result in the unnecessary distribution of mass antibiotic treatment. We aimed to evaluate the diagnostic accuracy of a prototype point-of-care (POC) test, developed for field diagnosis of ocular *C. trachomatis*, in low prevalence settings of The Gambia and Senegal.

**Methodology/Principal Findings:**

Three studies were conducted, two in The Gambia and one in Senegal. Children under the age of 10 years were screened for the clinical signs of trachoma. Two ocular swabs were taken from the right eye. The first swab was tested by the POC test in the field and the result independently graded by two readers. The second swab was tested for the presence of *C. trachomatis* by Amplicor Polymerase Chain Reaction. In Senegal, measurements of humidity and temperature in the field were taken. A total of 3734 children were screened, 950 in the first and 1171 in the second Gambian study, and 1613 in Senegal. The sensitivity of the prototype POC test ranged between 33.3–67.9%, the specificity between 92.4–99.0%, the positive predictive value between 4.3–21.0%, and the negative predictive value between 98.0–99.8%. The rate of false-positives increased markedly at temperatures above 31.4°C and relative humidities below 11.4%.

**Conclusions/Significance:**

In its present format, this prototype POC test is not suitable for field diagnosis of ocular *C. trachomatis* as its specificity decreases in hot and dry conditions: the environment in which trachoma is predominantly found. In the absence of a suitable test for infection, trachoma diagnosis remains dependent on clinical signs. Under current WHO recommendations, this is likely resulting in the continued mass treatment of non-infected communities.

## Author Summary

Trachoma, caused by infection of the eye with the bacterium *Chlamydia trachomatis*, is the leading infectious cause of blindness and is associated with poverty. Antibiotic treatment of all community members is one of the recommended control strategies for trachoma. However, in places where the prevalence of clinical signs is low, *C. trachomatis* eye infection is often absent. Laboratory testing for *C. trachomatis* infection by polymerase chain reaction (PCR) is highly sensitive but expensive and requires well-trained staff. A simple point-of-care (POC) test that can be used in trachoma-affected communities could help trachoma control efforts. We evaluated a POC test for *C. trachomatis* eye infection. Children under 10 years of age were screened for clinical signs of trachoma and *C. trachomatis* eye infection. The POC test result was compared with laboratory PCR test results. The POC test detected just over half of PCR test positives correctly. However, the POC test tended to give false-positive results in hot and dry conditions, which is the typical environment of trachoma. The POC test requires high specificity since it would be used to make treatment decisions at the community level. Therefore, its present format requires improvement before it can be utilized in trachoma control.

## Introduction

Trachoma is caused by ocular infection with the bacterium *Chlamydia trachomatis* and is the leading infectious cause of blindness worldwide [1]. The World Health Organization (WHO) simplified grading system, designed for the simple and reliable grading of trachoma clinical signs by non-specialist staff, is predominantly used for trachoma diagnosis in the field [2]. This system classifies the clinical signs into five categories: trachomatous inflammation-follicular (TF), trachomatous inflammation-intense (TI), trachomatous scarring (TS), trachomatous trichiasis (TT), and corneal opacity (CO).

Clinical signs are however poorly correlated with detection of ocular *C. trachomatis* infection, since they may persist for months or years after infection has cleared [3], [4], [5], [6], [7]. The WHO recommends that any district or community where the prevalence of TF in children aged 1–9 years is at least 10% should receive mass antibiotic treatment annually for three years, before the prevalence is re-assessed [8]. Since antibiotics are given to treat *C. trachomatis* infection, and the prevalence of clinical signs is a poor predictor of infection especially in low prevalence and mass treated settings, treatment may be unnecessarily commenced and continued, thus wasting scarce resources. A point-of-care (POC) test capable of detecting infection in the field would enable treatment to be directed to those communities in need. Since a POC test would be used to make treatment decisions at the community, rather than the individual, level, it is important that it has high specificity (>98%), otherwise it has no advantage over the use of clinical signs.

A prototype POC test for trachoma, developed by the Diagnostics Development Unit (University of Cambridge, UK), and currently not commercially available, has previously been evaluated on a small scale in a medium prevalence Tanzanian setting (12.5–37.9% TF in children aged 1–9 years), with encouraging results [9]. This assay is a modified version of a test for genital *C. trachomatis* infection [10], [11], optimised for use with conjunctival swabs. The assay detects the chlamydial lipopolysaccharide (LPS), using lateral flow technology. The dipstick is made up of a nitrocellulose membrane affixed to a backing sheet, and connected to an absorbent pad, with two immobilised monoclonal antibodies (mAbs) lined on the dipstick membrane. The mAb at the capture line is against chlamydial LPS, and that at the procedural control line is an antibiotin antibody. This assay was designed specifically for use in resource-limited settings, and therefore has no electricity, water or laboratory equipment requirements [9].

We aimed to conduct a larger scale evaluation of this prototype POC test's diagnostic accuracy in children aged under 10 years in the low prevalence settings of The Gambia and Senegal. The functional temperature and humidity range of the prototype test was unknown before this study's field testing.

## Methods

The study has been reported in accordance with the STARD (STAndards for the Reporting of Diagnostic accuracy studies) checklist (provided as Supporting Information S1) [12].

### Ethics statement

Research was done in accordance with the declaration of Helsinki. Ethical approval was obtained from the London School of Hygiene & Tropical Medicine (LSHTM) ethics committee (No.2067), the Gambia Government/Medical Research Council Joint Ethics Committee (SCC 979), and the Comité d'éthique du CNRS, Dakar, Senegal. Written (thumbprint or signature) informed consent was obtained from the guardians of all children.

### Study site and participant selection

**Figure 1:**
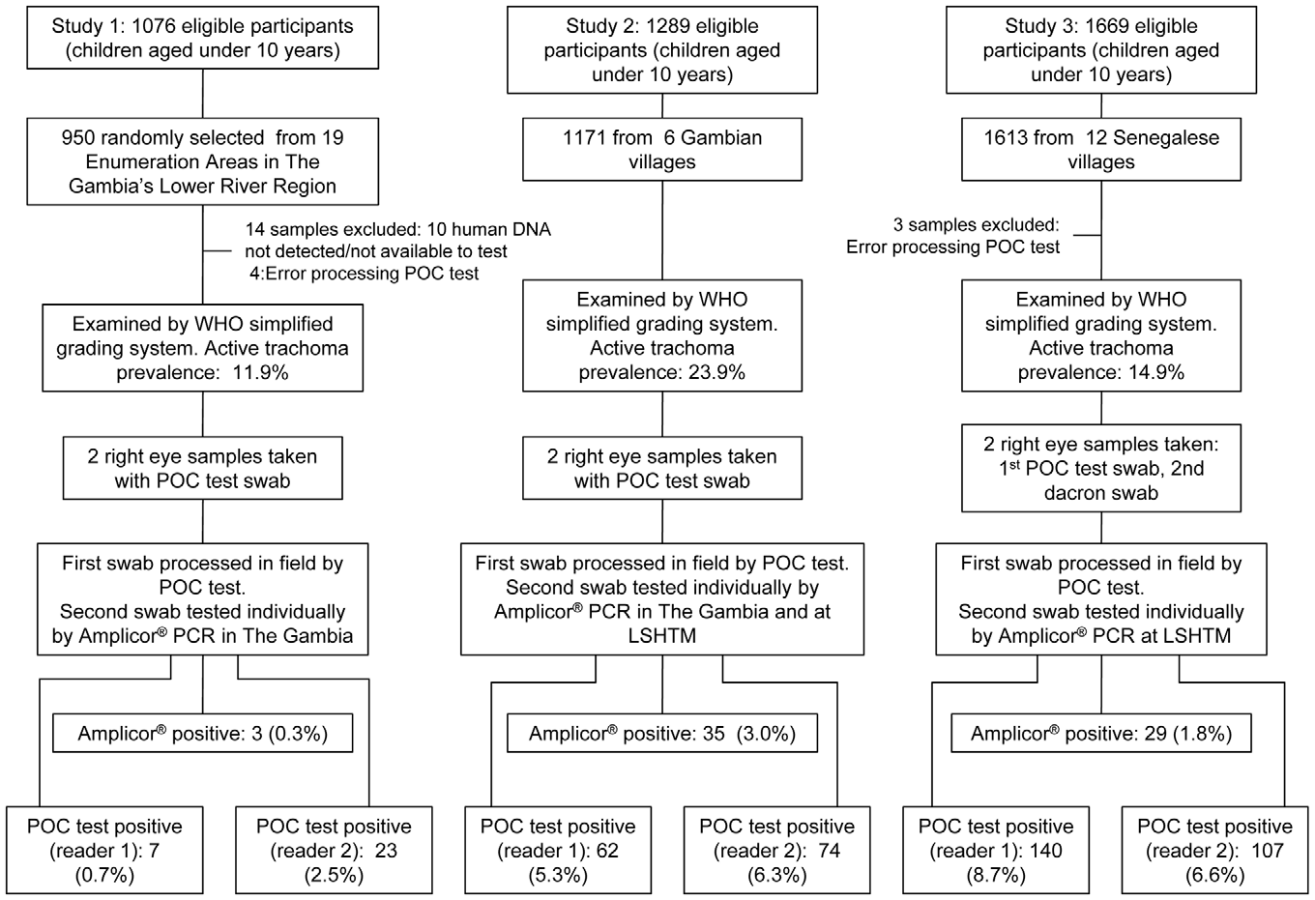
Flowchart outlining study methods and results.

Three studies were conducted, two in The Gambia and one in Senegal. An overview of the study methods is depicted in Figure 1. Study 1 was part of a survey of the Lower River (LRR) and North Bank (NBR) Regions of The Gambia. The sample selection has been described in detail elsewhere [13]. Briefly, 19 census Enumeration Areas (EAs), which are designed to be of approximately the same population size, were randomly selected in LRR. A random selection of households was made so that 50 children aged under 10 years would be included. In Studies 2 and 3, all children aged under 10 years were included. Study 2 took place in 6 Gambian communities and Study 3 in 12 Senegalese communities. The Gambian communities were selected on the basis of having a TF prevalence of at least 10% in the Gambian survey [13], increasing the likelihood of finding infection. Study 3 was based in the health post of Keur Samba Kane in Bambey District, which had been identified by the National Eye Care Programme as fulfilling the WHO criteria for mass treatment. Study 1 was conducted in January–March 2006, Study 2 in March–May 2006, and Study 3 in January–February 2007.

### Census enumeration

The village head (*alkalo*) and villagers were sensitised to the study's aims and methods. Household head lists were made and the *de facto* population was enumerated, recording their name, alias names, age and sex. Date-of-birth was noted when possible using ID cards and infant vaccination cards. The census team identified eligible children and informed household heads of the day and place of examination to ensure optimum participation.

### Clinical examination

Experienced Gambian and Senegalese graders were used. Their grading was verified and standardised using WHO grading slides, and a chance corrected agreement (Cohen's kappa [14]) score of at least 0.8 was required for the scoring of each sign (TF, TI, TS, TT). NBR villages with the highest active trachoma prevalence in the Gambian survey were re-visited and children diagnosed with active disease were re-screened by a senior grader to verify clinical diagnoses.

The examination team located itself in a central point in the village. Eligible children were called and written informed consent (signature or thumbprint) from the participants' guardians was obtained. The validated grader examined each consenting participant's eyes using a 2.5× magnifying loupe and torchlight. In order to avoid cross-contamination, the examiner wore and changed gloves between each participant. The clinical diagnosis was made according to the WHO simplified grading system [2].

### Ocular sample collection

Two swabs were taken from the tarsal conjunctiva of each participant's right eye using a standardised technique [15], whereby the swab was held horizontally and drawn lengthways across the everted upper tarsal conjunctiva four times, rotating the head of the swab a quarter turn with each pass. Cross-contamination of samples was limited by using a field worker to pass the swab to the examiner. The field worker then held the tube into which the swab would be stored dry, so that the examiner never touched the tube, and the swab's head only ever contacted the participant's conjunctiva.

In The Gambia, both samples were collected with the POC test's sterile polyurethane swab (Becton, Dickinson and Company, Franklin Lakes, USA). In Senegal, the first sample was collected with the POC test's swab, and the second sample was with a dry Dacron polyester-tipped swab (Quelab Laboratories, Montreal, Canada). This swab change was because inhibition in Studies 1 and 2 was believed to be due to the polyurethane swab, as a cloudy lysate was observed in the Amplicor extract.

The first swab was processed immediately in the field by the POC test. The second-collected swabs, to later be tested for the detection of ocular *C. trachomatis* with the qualitative PCR Amplicor *Chlamydia trachomatis/Neisseria gonorrhoeae* (CT/NG) Test (Roche Molecular Systems, Indianapolis, IN, USA), were stored in a cool box in the field and archived frozen at −20°C within ten hours of collection.

### POC testing

POC testing was carried out according to the POC test's protocol [9]. Briefly, the eye swab was placed in a sample preparation tube to which three reagents were added for the release of *Chlamydia* LPS. Five drops of the sample extract were transferred to a detection tube, rehydrating two lyophilised signal amplification reagents. A dipstick was then placed inside the tube and the mixture was left to wick up for 25 minutes before the results were read.

The same person performed all POC testing and was masked to the clinical diagnosis. Results were read at 25 minutes by two different readers each masked to the other's grading. The first reader was trained by the Diagnostics Development Unit, and the second reader was trained by the first reader. Grading was practised on non-clinical samples prior to participant sample collection. The signal strength was graded from 0 (negative) to 5 (strongly positive) using a signal grading card with increments of 0.5. A positive sample is defined as any signal with a signal strength of 0.5 or more noted by the reader. In Senegal, a pocket size temperature/humidity handheld datalogger (RH32 Series, Omega, Manchester, UK) was used with values measured every 30 minutes.

### Amplicor PCR processing

Amplicor, which detects the multi-copy cryptic plasmid, was performed on the second-collected swab. Amplicor was chosen as the reference test due to its good diagnostic performance on ocular samples [16], [17], [18], its history of use for detection of ocular *C. trachomatis* detection [15], [19], [20], [21], and its use as the reference test in the previous evaluation of this prototype POC test [9].

Study 1 samples were tested within 42 days of collection at the Medical Research Council (MRC) Laboratories, Fajara, The Gambia. About half of Study 2 samples were processed within 1 month at the MRC, and the remainder within 4 months at the London School of Hygiene & Tropical Medicine (LSHTM). All Senegalese samples were processed at the LSHTM between 2 and 6 months of collection. A previously published [15] sample preparation protocol was used instead of that in the Amplicor package insert. Positive and negative controls provided with the assay were included to validate the runs. When clusters of positives were observed on the detection plate, the positive samples were retested on-site. Those confirmed positive on the retest were considered Amplicor positives, and the others were considered negatives. Amplification of both the plasmid DNA and the master-mix internal control sequence was tested, allowing for inhibition to be detected. Inhibited samples were diluted from 1/5 up to 1/100 with a 50∶50 lysis∶diluent mix, until inhibition was resolved.

### Quantitative PCR processing

The bacterial load of Amplicor positive samples was estimated by processing the samples with a real-time quantitative PCR assay targeting the single-copy *ompA* gene [15]. The reverse primer, common to all ocular serovars, was 5′-TTT AGG TTT AGA TTG AGC ATA TTG GA-3′. The serovar A and B forward primers were 5′-GCT GTG GTT GAG CTT TAT ACA GAC AC-3′ and 5′-TCT GTT GTT GAG TTG TAT ACA GAT AC-3′ (Sigma-Genosys, Gillingham, UK), respectively. Quantitation was done on two 4 µL replicate samples for both serovar A and serovar B. The Gambian samples were processed in a LightCycler (Roche Diagnostics, Indianapolis, USA). The Senegalese samples were processed on a Rotor-Gene RG3000 (Qiagen, Crawley, UK).

### Quality control

Protocol changes were introduced as the study progressed to help ensure data quality. These changes involved the introduction of a POC test panel to be performed in the field, mock swabs inserted between patient samples in the field, environmental controls (air, loupe and glove swabs), and testing for laboratory contamination.

#### Panel

For Studies 2 and 3, a panel of positive (low, medium and high concentrations of *C. trachomatis* LGV-L1) and negative controls was processed at the beginning of each working morning and afternoon to check that the POC test was working correctly. A swab was dipped into the control vial (for the negative control: 10 mM PBS, 0.1% sodium azide and 1% treated casein; concentrations for the positive controls which should all be POC test positive: High: 1.143×10^7^ *C. trachomatis* elementary bodies (EB)/ml; Medium: 9×10^5^ EB/ml; Low: 2.25×10^5^ EB/ml) and processed normally. Before the addition of the third reagent, 50 µL of these controls was aliquoted into 200 µL of pre-dispensed Amplicor diluent to be later processed by Amplicor, to serve as negative and positive controls from the field to the laboratory. These aliquoted panels were stored in the same way as the ocular swabs.

In Senegal, two types of field sample control were introduced:

#### Environmental controls

At approximately every hundredth sample, a swab was waved in the air (air control), another swab wiped the top of the glove box (glove control), and a third swab wiped the front of the loupe (loupe control).

#### Mock swabs

Pre-prepared mock swabs were introduced between patient samples in the field before POC testing. Positive swabs were prepared with a non-ocular *C. trachomatis* strain (LGV-L1). The low load positive was below the POC test detection limit (2500 chlamydial EBs per test [9]) and the high load positive was above the detection limit. Negative mock swabs were also included. Mock swabs were introduced at a frequency which meant that one of each type of specimen would be processed per 89 patient samples. The samples were labelled in the same way as normal swabs to mask the POC and Amplicor test processors.

#### Amplicor quality control

At both LSHTM and MRC, Amplicor was conducted by experienced laboratory staff who had successfully completed a masked panel of samples. The Amplicor processors were masked to the clinical diagnoses and POC test results. As a measure of Amplicor reproducibility, the Amplicor-positive samples from Study 2 (20 tested at MRC, 15 tested at LSHTM) were re-tested by Amplicor at the University of Cambridge, and 21 Amplicor-negative samples were re-tested at LSHTM by a scientific officer who did not know the samples' origin. As part of the ongoing improvement to quality assurance throughout the study, it was decided to check for laboratory contamination at the time of processing the Senegalese samples. Swabs were taken of the laboratory cabinet surfaces and gloves, and processed by Amplicor.

To verify the quality of sample collection, Study 1 samples were tested for the presence of human-specific hypervariable 1 (HV1) D-loop region mitochondrial DNA (mtDNA) [13]. mtDNA-positive results indicate that human DNA is present in the sample.

### Statistical analyses

Results were double-entered by different entry clerks and verified in Microsoft Access (MS Access v2000/2003XP). Any discrepancies after verification were checked against the original paper forms. Data cleaning was performed in Stata (v9.2, STATA Corp., College Station, TX, USA).

Data analysis was performed in Stata, except for the humidity and temperature analyses which were performed in R (v 2.9.0, R Foundation for Statistical Computing, Vienna, Austria). As a result of the change in Amplicor swab type between Studies 2 and 3, and that the graders in The Gambia and Senegal were different, results from the 3 studies have not been combined.

The kappa statistic was used to assess between-grader agreement for the POC test and to assess Amplicor reproducibility. The performance (sensitivity, specificity, Positive Predictive Value (PPV) and Negative Predictive Value (NPV)) of the POC test was compared against Amplicor as the gold standard. Binomial exact 95% confidence intervals (CI) were calculated to quantify uncertainty. Proportions were compared using Pearson's chi-squared statistic. Cuzick's trend test was used to look at the relationship between quantitative load, clinical sign status and POC test result.

**Figure 2:**
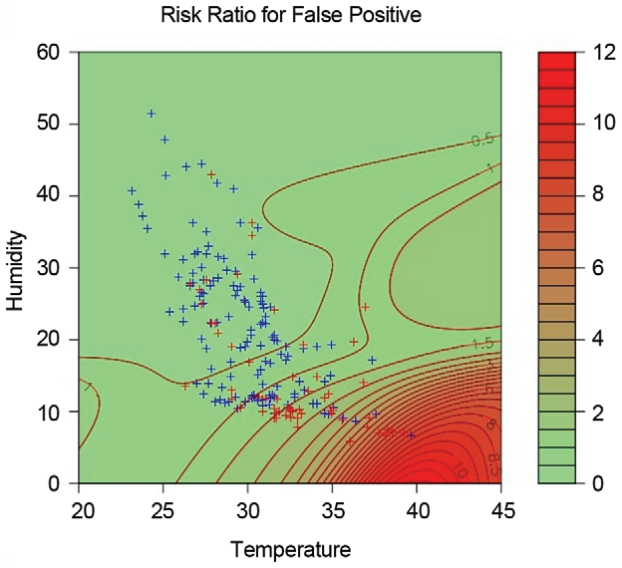
Relative risk of false-positives relative to true-negatives by temperature and relative humidity for Study 3.

The effect of temperature and humidity on the POC test's performance was measured using logistic regression. A scatter plot of false-positives (FPs) and true-negatives (TNs) by temperature and humidity was made, with contours of the relative risk of FPs relative to TNs. For each TN, a bivariate Normal density function was centred on the corresponding point. At any point on the graph, a density for TNs was calculated by summing these individual densities. A similar procedure was applied to the FP results. At any point, the relative risk is the ratio of these two densities. Contours of this relative risk were then added to the scatter plot (Figure 2).

## Results

A total of 3734 children were screened and tested. Study 1 (LRR, The Gambia) included 950 children under 10 years, Study 2 (6 villages, The Gambia) 1171, and Study 3 (Senegal) 1613. This represents participation of 88.3% (950/1076), 90.8% (1171/1289), and 96.6% (1613/1669) based on the censused population in Studies 1, 2, and 3, respectively.

### Laboratory controls

During Amplicor processing of the Senegalese samples, 12 lab controls were taken to check for lab contamination (3 for the hood, 2 of the glove box, 2 of the cabinet, and 5 of the gloves). All were Amplicor-negative.

### Mock swabs

In Senegal, 14 negative, 15 low load, and 11 high load mock swabs were introduced in between patient samples. Amplicor correctly detected all results. For the POC test, the number of correctly identified negative, low and high load positive cases differed significantly for reader 1 (p = 0.007) and reader 2 (p = 0.004). For both readers, the POC test correctly detected the high load positives in 100% of cases. These had a signal strength ranging from 1.0 to 2.0 for reader 1, and from 1.0 to 2.5 for reader 2. For low load positives (that tested POC negative under standard laboratory conditions), 5 samples were graded as positive by both the first and second readers. An additional sample was graded positive by reader 1, and 3 other samples as positive by reader 2. Thus, a total of 9/15 low load positives were detected by the POC test in the field. The signal strength of these false-positives ranged from 0.5 to 1.5 for both readers. For the negative controls, the readers both graded 5 samples as positive, and reader 2 additionally graded 4 samples as positive. These false-positives had signal strengths of 0.5 or 1.0 for both readers.

### Environmental controls

In Senegal, there were 16 air controls, 16 glove controls, and 17 loupe controls. All were Amplicor negative. Less than half the POC test results were negative for both reader 1 (42.9%) and reader 2 (46.9%).

### POC test panel

In total, there were 101 panel positive and negative controls aliquoted in the field and tested by Amplicor (52 in Study 2 and 49 in Study 3). All 63 positive controls were correctly detected by Amplicor. Of the 38 negative controls, one from The Gambia tested positive repeatedly and two initially tested equivocal but were negative when repeat tested in duplicate. There was an additional equivocal negative control result by Amplicor, but the sample was erroneously labelled only as “negative control” on the template, without specifying which negative control this was, meaning it could not be retested.

A total of 56 panels were tested by the POC test (14 in Study 2 and 42 in Study 3). All positive panels, regardless of concentration, were positive by the POC test for both readers. The proportion of all negative panels correctly recorded as negative by the POC test was 60.7% for reader 1 (85.7% for Study 2, 52.4% for Study 3), and 66.1% for reader 2 (92.9% for Study 2 and 57.1% for Study 3).

### Sample quality

Of 942 Amplicor-negative samples for which sample was available in Study 1, positive results for human-specific hypervariable D-loop region mtDNA were obtained in 937 (99.5%) samples. The five mtDNA-negative samples and five samples that could not be tested for mtDNA because of insufficient material from Study 1 have been removed from analyses. Three field air controls were randomly selected and also tested for *C. trachomatis* and human mtDNA, and provided negative results.

### Inhibition by swab type

Inhibition in Studies 1 and 2 which used the POC test polyurethane swab was 23.4% (220/940) and 22.8% (2671171), respectively. The proportion of inhibited samples in Study 3 was 18.2% (293/1613), so the change of swab did not make a noticeable difference to the level of inhibition. Only one inhibited sample, from Study 2, retested as Amplicor positive.

### Amplicor reproducibility

Of the 35 Amplicor-positive samples from Study 2 retested at the University of Cambridge, 27 were confirmed positive (23 as positive and 4 as equivocal), 3 were negative but failed the Internal Control (IC), and 5 were negative and passed the IC. All 21 Amplicor-negatives retested as negative. These retests resulted in a kappa score between the initial and retest results of 0.73, demonstrating substantial agreement.

All 35 samples originally tested as positive were considered true positives for the analyses presented. Of the 5 negatives, three were positive by quantitative PCR with estimated loads of 5, 7 and 25 *ompA* copies/swab. The remaining two positives were isolated among a string of negatives in the field, and were not near positive samples on the Amplicor detection plate.

If a true positive was considered to be one that was positive at both LSHTM and the University of Cambridge (27 samples retested positive or equivocal), the specificity and PPV estimates remain the same. The NPV increases slightly to 98.7% (97.9–99.3) for reader 1 but decreases to 98.8% (98.0–99.4) for reader 2. The sensitivity increases for both readers, but insignificantly: 48.1% (28.7–68.1, p = 0.384) for reader 1 and 51.9% (31.9–71.3, p = 0.352) for reader 2.

### Repeat of Amplicor positive samples clustered on the detection plate

For Study 1, none of the 3 Amplicor positives were retested. For Study 2, 10/39 Amplicor positives were retested, and 6 retested negative. For Study 3, 13/51 Amplicor positives were retested and all retested positive.

### Diagnostic performance

The prevalence of active trachoma and Amplicor positives was, respectively, 11.9% and 0.3% in Study 1, 23.9% and 3.0% in Study 2, and 14.9% and 1.8% in Study 3. During field processing of the POC test, mistakes were made for 4 samples in Study 1, and 3 samples in Study 3. These samples have been removed from analyses involving the POC test. The POC test's sensitivity, specificity, PPV and NPV against Amplicor showed similar point estimates and 95% CI for the two readers (Table 1). Overall, sensitivity and PPV were low, with respective estimates ranging from 33.3%–67.9%, and 4.3%–21.0%. The specificity met the minimum target of 98% in Study 1, but not in Studies 2 or 3.

**Table 1:** Diagnostic accuracy of the POC test compared with the gold standard (Amplicor).

	PCR	POC test - reader 1			POC test - reader 2		
		Negative	Positive	Total	Negative	Positive	Total
Study 1	Negative	927	6	933	911	22	933
	Positive	2	1	3	2	1	3
	Total	929	7	936	913	23	936

		Negative	Positive	Total	Negative	Positive	Total
Study 2	Negative	1087	49	1136	1076	60	1136
	Positive	22	13	35	21	14	35
	Total	1109	62	1171	1097	74	1171

		Negative	Positive	Total	Negative	Positive	Total
Study 3	Negative	1461	121	1582	1492	90	1582
	Positive	9	19	28	11	17	28
	Total	1470	140	1610	1503	107	1610

There is no evidence of a significant difference between the point estimates and corresponding 95% CI for NPV, PPV, or sensitivity between the three studies. Precision for the sensitivity estimates was low due to small numbers (Table 1). Compared with Study 1, the specificity of the POC test was significantly lower in both Study 2 (p<0.001) and Study 3 (p = 0.001). In Studies 2 and 3, the specificity upper confidence bounds did not exceed 96.8%.

### Effect of relative humidity and temperature on diagnostic performance

Temperature and relative humidity data were collected for all samples from Study 3 (1584 Amplicor-negatives and 29 Amplicor-positives). Figure 2 shows contours of the relative risk (RR) for FPs relative to TNs, with shading from green to red as the RR increases (see under Statistical Analyses in the Methods section). It is apparent that the false-positive RR began to increase at temperatures above 30°C and at relative humidities below 10%. The RR of a FP is approximately three times that of a true-negative at a temperature of about 36°C and at a relative humidity of 10%, and increases more rapidly as temperature rises and humidity falls.

Plots of FP rates against temperature and relative humidity indicated an increase at temperatures above 31.4°C, and a relative humidity below 11.4%. Estimates of diagnostic accuracy calculated for samples processed above and below the 31.4°C temperature threshold showed that the specificity was significantly lower in samples processed above the threshold than below, for both the first and second POC test readers (p<0.001) (Table 2). For humidity, the specificity was significantly lower in samples processed below a threshold of 11.4% compared with those above the threshold, for both readers (p<0.001) (Table 2).

**Table 2:** Effect of temperature and humidity on performance of the POC test compared to Amplicor PCR.

Temperature threshold: 31.4°C				
	POC test – reader 1		POC test – reader 2	
	Below threshold	Above threshold	Below threshold	Above threshold
True positive	10	9	10	7
True negative	956	505	961	531
False positive	21	100	16	74
False negative	7	2	7	4
Sensitivity	58.8(32.9–81.6)	81.8(48.2–97.7)	58.8(32.9–81.6)	63.6(30.8–89.1)
Specificity	97.9(96.7–98.7)	83.5(80.3–86.4)	98.4(97.4–99.1)	87.8(84.9–90.3)
PPV	32.3(16.7–51.4)	8.3(3.8–15.1)	38.5(20.2–59.4)	8.6(3.5–17.0)
NPV	99.3(98.5–99.7)	99.6(98.6–100)	99.3(98.5–99.7)	99.3(98.1–99.8)

Relative humidity threshold: 11.4%				
	POC test – reader 1		POC test – reader 2	
	Below threshold	Above threshold	Below threshold	Above threshold
True positive	6	13	5	12
True negative	381	1080	403	1089
False positive	90	31	68	22
False negative	2	7	3	8
Sensitivity	75(34.9–96.8)	65(40.8–84.6)	62.5(24.5–91.5)	60(36.1–80.9)
Specificity	80.9(77.0–84.3)	97.2(96.1–98.1)	85.6(82.1–88.6)	98(97.0–98.8)
PPV	6.3(2.3–13.1)	30.2(17.2–46.1)	6.8(2.3–15.3)	35.3(19.7–53.5)
NPV	99.5(98.1–99.9)	99.4(98.7–99.7)	99.3(97.9–99.8)	99.3(98.6–99.7)

Values in parentheses are 95% CI.

### Analytic sensitivity of the POC test

Of the 67 Amplicor-positives, positive *ompA* results were obtained in 58 (86.6%) samples by quantitative PCR. The estimated number of *ompA* copies/swab ranged from 5 to 3,008,063, with a median of 670. Although a few low load PCR positives were POC test positive, the POC test consistently detected positives from 1000 *ompA* copies/swab. Although the POC test is a qualitative assay, the signal strength was scored on a scale from 0.5 (weak) to 5.0 (strong) in the field. There was a significant association between increased organism load and increased POC test signal strength (p<0.001).

### Inter-grader agreement

The kappa score for inter-grader variability between the two POC test readers was lowest for Study 1 and highest for Study 3. For exact signal strength the kappa score ranged from 0.41 to 0.59, showing moderate agreement. When the results were categorised as positive (signal strength ≥0.5) or negative (signal strength <0.5), the scores ranged from 0.26 to 0.68, demonstrating fair to substantial agreement.

## Discussion

In this study we conducted an evaluation of a prototype POC test for the detection of ocular *C. trachomatis* in children aged under 10 years in The Gambia and Senegal. After following standardised field and laboratory protocols, ensuring quality assurance and data validity, the results demonstrated that in its present format, this POC test is not suitable for use in the field. Under laboratory conditions, the negative and low positive mock swabs resulted in negative POC tests. In the field, the POC test gave false-positive results for approximately half of these mock swabs. This demonstrates that the POC test does not pass quality control procedures when tested in the field. When tested on children's ocular swabs, specificity in Study 1 was excellent (99.0% and 97.6% for readers 1 and 2, respectively). This is consistent with the specificity reported from the previous evaluation of this test performed in Tanzania, where the overall specificity was 99.4% (95%CI 98.8–100) [9]. However, in Studies 2 and 3, the specificity ranged from 92.4% to 95.7%, falling short of the 98% minimum specificity required for this test.

The temperature and relative humidity data provide the most likely explanation for the lower POC test specificity in Studies 2 and 3. Study 1 was conducted in January and February, when The Gambia is experiencing its cool season. Study 2 took place just before the rainy season, when temperatures rise. In Study 3, high temperatures and low relative humidities were recorded whilst performing the test, and these conditions were shown to significantly affect the false positive rate of the POC test. These data indicate that the prototype POC test's format is not appropriate for these environmental conditions.

Evaluations of rapid POC tests for other infectious diseases have observed a detrimental effect of high temperature and humidity during test storage on performance [22], [23], [24]. However, we observed an effect on the test's performance in the field during processing. A review of malaria rapid diagnostic tests which also use lateral flow technology, notes that humidity and wind rapidly degrade nitrocellulose capillary flow action. This effect on reagent flow could result in false-positives. In addition, temperature and time could be detrimental to the test's sensitivity, as they have been reported to deconjugate the signal line antibody-indicator complex, detach the capture antibody from the nitrocellulose strip, and unfold the binding sites of antibodies [25]. Since the dipstick of the ocular *C. trachomatis* POC test under evaluation is made up of a nitrocellulose membrane transversely lined with mAb against chlamydial LPS and antibiotin, these are plausible explanations for the observed deleterious effect of high temperature and low relative humidity on the test's performance. These results suggest that the environmental conditions during Studies 2 and 3 were harsher than those experienced in Study 1 and Tanzania, and emphasise the importance of conducting POC test evaluations in different settings. A change in the format of the prototype POC test that prevents its performance from being affected by the dry, hot, and dusty environments in which trachoma is predominantly found [26], would no doubt improve the usefulness of this test for trachoma control.

False-positives may also have appeared as a result of the POC test's target being the genus-specific chlamydial LPS. We do not believe, however, that cross-reaction with non-*C. trachomatis* bacteria was the cause of the POC test false-positives observed in this study. As noted by Michel *et al.*, the POC test's specificity has been established against a panel of microorganisms commonly associated with the human eye and skin (such as *Staphylococcus*, *Pseudomonas*, *Streptococcus*, *Escherichia*, *Proteus*, and *Candida*, obtained from ATCC) [9]. In addition, if cross-reaction were taking place, it would not explain the observed association between FPs with temperature and humidity.

The advantage of testing the prototype POC test in low prevalence settings was the ability to gain a good estimate of specificity. The disadvantage is that we have been unable to determine an accurate estimate of the test's sensitivity. In addition, the active disease found in this study was mild with only 6.5% of clinically active children having TI. Infection load is correlated with disease severity [4], [15], [27]. The consequence of lower infection loads is a lower test sensitivity, especially in an assay that detects a surface antigen as opposed to one using PCR technology. Indeed, the Tanzanian evaluation observed a lower (albeit non-significant) sensitivity (76.9%) of the POC test in the lower prevalence site (TF prevalence 12.5%) compared with a sensitivity of 85.5% where the TF prevalence was 31.5% [9]. Michel *et al.* (2006) noted that the assay has an analytical sensitivity of 2500 chlamydial EBs per test [9]. Our quantification demonstrated consistent detection from approximately 1000 *ompA* copies/swab.

In terms of the limitations of this study, there was a delay of up to 6 months between sample collection and sample processing, which could have resulted in low load positives testing negative. This may have contributed to the number of POC test false-positives observed. However, since samples were stored at −20°C, we do not believe that the DNA would have degraded and that waiting would have led to a decrease in the number of true positives.

The POC test was performed on the first-collected swab whereas the “gold standard” Amplicor testing was on the second-swab. There may be differences between the two swabs, for example, in cases where there are few EBs in the conjunctiva the first swab may not leave any for the second swab. One of the swabs may also collect more PCR-inhibiting material, such as mucous, resulting in inhibition in one of the assays. Furthermore, one swab may be passed more forcefully over the conjunctiva, collecting more DNA or inhibiting material. Michel *et al.* (2006) demonstrated that first-collected swabs had higher loads than second-collected swabs by comparing organism load in the first- and second-collected swabs from 13 Amplicor positive individuals. The first swab's mean EB count was 643,424 compared with 181,310 for the second swab. This should not affect the Amplicor prevalence as its detection level is in the range of 1–10 EBs [28], [29]. Furthermore, Amplicor result concordance between first- and second-collected swabs has been shown to be excellent [5], [30].

There was a change in swab type between Study 2 and Study 3 because it was believed that the polyurethane swab led to inhibition. However, the swab change did not make a noticeable difference to the level of inhibition. The disadvantage of inhibition is the need to dilute the sample, which would reduce the copy number in any sample tested, resulting in Amplicor false-negatives. Since load of infection in the study sites was often low (with 37.3% of all Amplicor positives having a load of <10 *ompA* copies/swab or being negative), this is a distinct possibility, and could have contributed to the low specificity of the POC test.

Another possible limitation is our choice of gold standard. In the absence of a universally accepted gold standard for *C. trachomatis*, we chose Amplicor as it was used in the previous evaluation of this POC test [9], and it has been used in multiple studies of ocular *C. trachomatis* infection. Controls included to assure the quality of our gold standard produced excellent results. Air, loupe, glove and spiked mock swab field controls were all correctly identified. The Amplicor results for aliquots from the POC test control panel were correct except for one negative panel from Study 2, which was repeatedly positive, and one equivocal which could not be repeat tested because the sample name was not correctly written on the Amplicor plate template. This suggests contamination of the negative panel from the positives when aliquoting in the field, which is possible as stringent laboratory conditions cannot be maintained in such an environment. Furthermore, it was a requisite for a successful run that the Amplicor-provided positive and negative controls processed for each plate produce the correct result, indicating that contamination in the lab is unlikely. This is supported by the Amplicor negative results of swabs taken of lab surfaces to check for lab contamination. When positives clustered on the detection plate were repeat tested, 6/10 retested samples from Study 2 retested negative. This could indicate that there was contamination between the wells on the detection plate, and for this reason they were considered negative in analyses. Alternatively, these samples could have been low load positives that did not repeat test positive. Of the 35 Amplicor positives retested by Amplicor at the University of Cambridge, five tested negative.. The failure to retest these five samples as positive was not unexpected as reproducibility when retesting the original sample with the same test is known to be poor for low load samples [31], [32], [33], [34], [35], [36]. However, when samples that were not repeated positive at the University of Cambridge were removed from the analyses, there was no significant effect on the prototype POC test's performance.

The development of effective diagnostic tools is considered a priority for Neglected Tropical Diseases (NTDs) [37], and it is therefore important to be aware of the impact the environment can have on the operational performance of POC tests. A lateral flow platform in an open system appears not to be suitable for the environments in which NTDs, such as trachoma, are often found. A rapid, accurate, simple, and affordable POC test which can be performed in the field could be a great asset to trachoma control, particularly in low prevalence settings. The specificity of the test must be high (>98%) to prevent communities from being unnecessarily mass treated. The specificity of the prototype POC test evaluated in this study decreased as the temperature increased and relative humidity decreased, indicating the importance of field testing POC tests in the different environments in which the target disease is found, in addition to being evaluated in different prevalence settings. Until a suitable test is made available, trachoma control decisions in the field remain reliant on clinical diagnosis, potentially wasting scarce resources.

## Supporting Information

Supporting Information S1 **STARD checklist.**
(DOC)
