# Supplementary material for: Diagnostic Accuracy of a Prototype Point-of-Care Test for Ocular Chlamydia trachomatis under Field Conditions in The Gambia and Senegal
Source: PLoS Negl Trop Dis. 2011 Aug 2;5(8):e1234. doi: 10.1371/journal.pntd.0001234 (PMC3149007; doi:10.1371/journal.pntd.0001234)
Supplement: Supporting Information S1 — STARD checklist. (DOC) [file pntd.0001234.s001.doc]

STARD checklist for the reporting of studies of diagnostic accuracy. 
First official version, January 2003. 
